# Molecular combing and its application in clinical settings

**DOI:** 10.1186/s13039-022-00628-8

**Published:** 2022-11-16

**Authors:** Yiping Wang, Kishore Ramesh Kumar, Thomas Liehr

**Affiliations:** 1 Institute of Human Genetics, Jena University Hospital, Friedrich Schiller University, Am Klinikum 1, 07747 Jena, Germany; 2 Jinze Group Beijing Jinze Medical Laboratory, Building A, National Engineering Center for Protein Drugs, Building 1, 33 Science Park Road, Huilongguan Town, Changping District, Beijing, 1F China

**Keywords:** Molecular combing, Fluorescence in situ hybridization (FISH), Genomics, Constitutional genetic diseases, Acquired genetic diseases

## Abstract

Molecular combing technology (MCT) is an effective means for stretching DNA molecules and making them thus accessible for in situ studies. MCT uses the force exerted in the process of liquid flow via surface tension to stretch DNA molecules and spread them on solid surfaces, i.e. glass cover slips. Many DNA molecules can be stretched at the same time in parallel and neatly arranged side-by-side, making the approach convenient for statistical analysis. Accordingly, DNA replication and transcription can be studied at the single molecule level. In this paper, the principle, experimental methods, important applications, advantages and shortcuts of MCT in medical field are presented and discussed.

## Background

Molecular cytogenetics is the study of genomic alterations based on techniques associated with in situ hybridization. In the 1980’s, fluorescence in situ hybridization (FISH) was developed from the radioactive variant of the technique and applied on human cytogenetic preparations [1]. At first, FISH seemed to be mainly useful to localize (human) genes; however, quickly the technology was adapted for clinical and tumor cytogenetics to characterize chromosomal rearrangements being unresolvable in banding cytogenetics (for review see [2]). At first, progress was driven by research-based laboratories, producing probes suited for FISH by cloning, glass-needle based chromosome microdissection or chromosome flow sorting [3]. These laboratories also introduced multicolor-FISH approaches like locus-specific probe based multiplex subtelomeric FISH [4], partial chromosome painting probe dependent multicolor banding (MCB) [5] or whole chromosome painting probe based spectral karyotyping (SKY) [6] and multicolor fluorescence in situ hybridization (M-FISH) [7]. Several of such probes and probe sets were also commercialized in parallel; for example MCB is available as mband-probe sets [8].

A limitation of chromosome-/metaphase oriented FISH is its power of resolution [2, 5]. Due to DNA compaction in metaphases it becomes difficult if not impossible to map the order of two or three genes along a chromosome if they are less than 2–5 Mb apart from each other [9]. In interphases DNA is more decondensed, still the order of three closely localized genes can only be determined reliably when evaluating 20–50 cells in a semi-statistical way; besides, the distance between them has to be in the range of 0.5 to 1 Mb or more. To achieve higher resolutions, approaches like fiber-FISH or molecular combing technique (MCT) were established [10–13]. In this review, MCT principle and how to perform, applications in medical field, advantages and shortcuts are presented and discussed.

## MCT–how it developed

The field of cytogenetics has focused in (i) medical genetics on studying the number, structure, function and origin of chromosomes and their abnormalities [2, 14], and (ii) in biology on the evolution of chromosomes [15]. The development of fluorescent molecules that either directly or via an intermediate-molecule bind to DNA [16] has led to the development of FISH, a technology linking cytogenetics to molecular genetics [2]. This technique has a wide range of applications that enlarged the possibilities of chromosome analysis [2]. The field of cytogenetics is particularly important for medical diagnostics and research as well as for gene mapping [2, 3]. Furthermore, the increased application of molecular biology techniques, such as array-based technologies, has led to improved resolution, extending the recognized range of microdeletion/microduplication syndromes and genomic disorders [17]. In adopting these newly expanded methods, cytogeneticists have used a range of technologies to study the association between visible chromosome rearrangements and defects at the single nucleotide level [18]. The development of molecular cytogenetic technology has increased the understanding of the possible molecular mechanisms involved in chromosomal rearrangements and genotype–phenotype associations, thereby helping patients to obtain better diagnosis and genetic counseling [2, 3].

FISH is a flexible technique that has driven the further development of different new molecular cytogenetic probe sets (see above) and/or applications. There are multiple approaches using FISH-based methods for different applications, like reverse-FISH [19], flow-FISH [20], Q-FISH (quantitative FISH) [21], cenM-FISH (centromere-specific M-FISH) [22], pod-FISH (parental origin determination FISH) [23], HCM-FISH (heterochromatin-oriented M-FISH) [24], and others. If modified, several FISH techniques can also be applied to interphase cells (interphase FISH) [25], which confers the advantages of FISH for the visualization of DNA probes in nuclei [26].

Different variants of FISH can be used to retrieve information on genomes from (almost) base pair to whole genomic level, as besides only second and third generation sequencing approaches can do [2]. Here especially to consider variations of FISH are chromosome orientation-FISH (CO-FISH) [27], Q-FISH [21], pod-FISH [22], FISH to resolve the nuclear architecture [9], multicolor-FISH approaches [2, 3], among other applied in chromoanagenesis studies [28] and MCT itself.

Fiber-FISH, also known as a MCT, hybridizes DNA probes to chromatin fibers stretched out on specimens, such as chromatin released from cells [10, 11]. An improved approach is to hybridize the probe with unfixed DNA fibers derived from cells embedded in pulsed-field gel electrophoresis clots. This method has been used for high-resolution gene mapping, gene replication, and direct observation of chromosomal breaks involved in translocations (see below for more details).

**Fig. 1:**
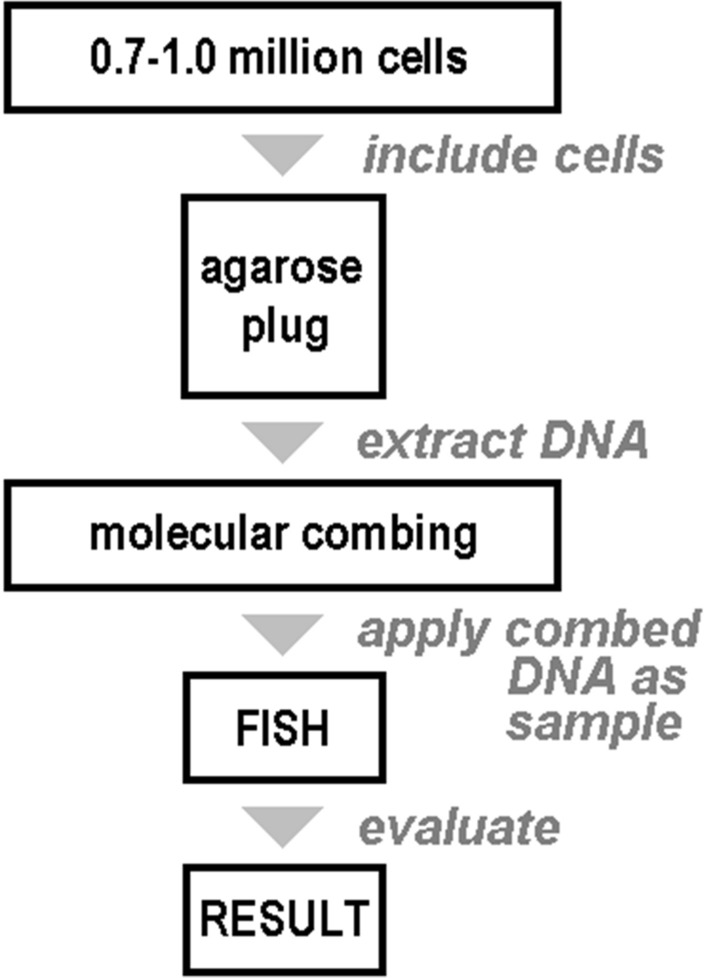
Scheme of molecular combing technique (MCT): 0.7 to 1 million of cells (either from cell culture or from peripheral blood lymphocytes) must be included in the experiment. Cells are collected and included in an agarose plug from which high molecular weight DNA is extracted. The latter can be applied for MCT itself and coverslips with DNA-fibers are produced. Coverslips with DNA-fibers (Fig. 2) can be used in standard FISH and obtained results can be evaluated using a fluorescence microscope

In 1994, Bensimon and coworkers [12] found that DNA could be uniformly straightened by a moving gas–liquid interface on a silanized substrate surface. They call this approach MCT, which can be used to straighten a large number of DNA molecules simultaneously and uniformly with a simple instrument (Fig. 1). As this procedure does not cause modifications in DNA sequence, it provides new possibilities to study the structure of DNA and especially the order of genes and loci. In the following substrate, straightening mechanism, pH condition, tension size were studied in detail to improve MCT [13].

As already implied before, MCT enables physical characterization of single genomes at the kilobase level of resolution over large genomic regions. An array of combed single DNA molecules is prepared by stretching molecules attached to a salinized glass surface with a receding air–water meniscus. By performing FISH on combed DNA, probe position can be directly visualized with respect to a closely located probe, enabling to construct physical maps and to detect micro-rearrangements (Fig. 1). Single-molecule DNA replication can also be monitored by detection of fluorophore labelled, incorporated nucleotide analogues on combed DNA molecules [29, 30]. Accordingly, problems to be solved in post genomic era can be faced thanks to MCT either via fluorescence (FM) and/or atomic force microscopy (AFM) [31].

### MCT—principle

MCT takes advantage of physical or chemical binding forces between a DNA molecule and a hydrophobic surface. A solution with pure and high molecular weight DNA being arranged in an irregular coil shape, contacts the coverslip surface due to Brownian molecular movement. After attaching there, DNA is stretched by the retreating liquid surface, so that it is neatly arranged on the solid surface—here DNA changes its conformation from irregular coil to linear shape, driven by hydrophobic and/or electrostatic force [12].

MCT includes the following four steps: preparation of

(i) Coverslips coated with a hydrophobic surfaces such as silane or polymethylmethacrylate and
(ii) A high concentration DNA solution; the latter is prepared by embedding of the cells from which DNA is to be extracted in agarose plugs. After enzymatic treatment and washing, the pure and long DNA fibers as needed are prepared.
(iii) Dipping and incubating the coated coverslip (from i) in the solution from (ii) for 5 min to bond the DNA to the coverslip.
(iv) Pulling out the coverslip of the solution (from i) at a certain speed. This is a most critical step and must be done at steady speed of optimally 300 µm/s with a constant stretching factor (1 mm = 2 kb) [32–34]. Air drying fixes the DNA fibers to the surface.

The obtained coverslips are hybridized with certain FISH-probes (according to the question to be studied) and then evaluated at FM or AFM. This can be done either manually, or by a scanner, where the results can evaluated statistically based on a special computer software (Genomic Vision, Bagneux, France) [32]. As the results obtained produce signal patterns of different lengths this kind of combination of “dashes and dots” is also referred to a „genomic Morse code “ (GMC) [35].

### Advantages and restrictions of MCT

**Fig. 2:**
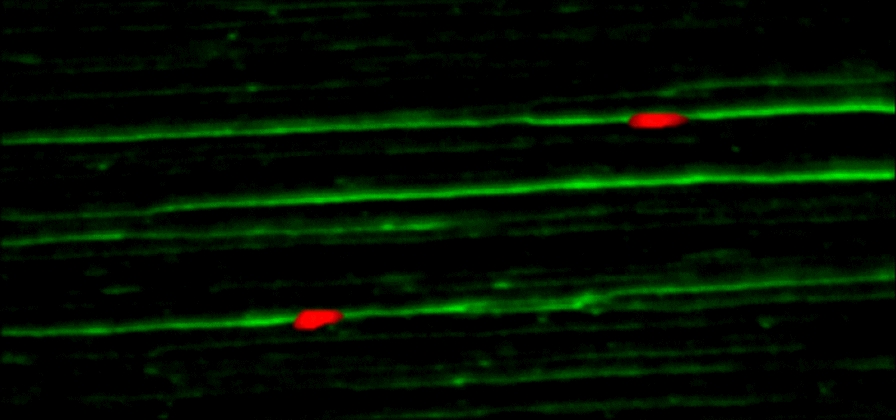
MCT result using the probe RP11-71J12 in 13q14.13 (GRCh37/ hg19; chr13:46,439,690–46,587,782) labeled in SpectrumOrange and hybridized to DNA fibers spread on homemade silanized coverslips [56]. DNA-fibers were stained by YOYO-1 (green). Picture was acquired on a Zeiss Axioplan microscope (Jena, Germany) using ISIS software (Metasystems, Altlussheim, Germany). The DNA fibers were produced using FiberComb – Molecular Combing System (Genomic Vision, Bagneux, France)

Clear advantages of MCT compared to other approaches is that it enables (a) visualization otherwise not accessible DNA-structures with (b) high sensitivity along single DNA-molecules of up to 12 Mb length [36]. (c) Regions from ~ 1 kb to 2 Mb can be studied applying FISH-probes which label 1 to 150 kb for deletions, duplications, amplifications and structural rearrangements, like inversions. (d) Results obtained are reliable and reproducible and MCT can accordingly be applied in clinical genetic diagnostics (see below). (e) As in other FISH based approaches multiplexing is possible, i.e. several loci can be accessed in parallel – only restriction are available fluorophores and number of filters in the detecting microscope (Fig. 2) or scanner [32].

Important limitations of MCT are, (A) that point mutations cannot be detected, (B) rearrangements below 1 kb in size may be missed, and (C) that commercial approach of MCT is limited to 2 to 3 probes, due to number of available detection filters in the commercially applied scanner [32].

### Possible clinical applications of MCT

MCT has principally opened up new possibilities to detect submicroscopic, but by sequencing hard to access, complex DNA abnormalities. The latter can be related to inborn or acquired genetic diseases as well as viral infection, and thus MCT has several (potential) clinical applications already, which are summarized below.

### MCT based clinical studies of gross chromosomal structures

While in plant genetics the possibilities to use MCT to characterize gross chromosomal structures (otherwise hard to access in detail) were already recognized early [33], in human such possibilities were only used occasionally. A literature review identified only three such studies: one being interested in the short arms of the acrocentric chromosomes and specifically the nucleolus organizing region [34], one characterizing a de novo microtriplication of 11q24.1 [35] and one to determine size of a microdeletion [36].

### MCT based studies of familial adult myoclonic epilepsy 1 and 3

Familial adult myoclonic epilepsy 1 and 3 (FAME1 and FAME 3 – OMIM #601,068 and #613,608) are autosomal dominant inherited syndromes, being characterized by adult-onset cortical tremor, and may be associated with seizures. In Chinese and Japanese populations FAME1 has been found to be caused by enlarged intronic TTTTA/TTTCA repeats in *SAMD12* gene in 8q24 [37, 38]. FAME3 is due to identical TTTTA/TTTCA repeat expansion in intron 1 of *MARCH6* gene in 5p15.2 [39]. MCT has been proven to be able to detect and quantify these repeat amplifications [32].

### MCT based diagnostics of facioscapulohumeral muscular dystrophy 1

Facioscapulohumeral muscular dystrophy type 1 (FSHD1- OMIM #158,900) is a disorder of skeletal muscles and shows (sometimes even within families) an extremely variable phenotype. In FSHD1, belonging to the group of hereditary progressive skeletal muscle dystrophies, a partial deletion of the D4Z4 repeats in 4q35 affects expression of *DUX4* gene, as one copy of this gene can be found within each D4Z4 repeat [40]. Standard molecular diagnosis relying on Southern blot can be challenging because D4Z4 stretches are also present in 10q26. Nonetheless, by MCT D4Z4 comprising regions on chromosome 4 and 10 can be visualized separately; in contrast to other approaches MCT also enables clearly distinguishing of D4Z4 stretches on each individual chromosome 4 and 10 [41]. Thus, the CE (Conformitè Européenne) certification for in-vitro diagnostics for an MCT based FSHD diagnostic assay was assigned to Genomic Vision, recently [32].

### MCT based studies in cancer

In diagnostics of tumors, single-molecule methods can help to detect and study large DNA rearrangements that lead to cancer [42].

- MCT based studies in leukemia

In a proof of principal study in 2016 Ittel and coworkers [43] showed, that MCT is well suited to identify variant breakpoints in “standard translocations” being associated with specific leukemia. Deviating breakpoint could be detected for translocation t(12;21)(p13;q22) involving *ETV6* and *RUNX1* genes, being typical for B-cell lineage childhood acute lymphoblastic leukemia.

- MCT based studies of BRCA1 gene

A certain subset of hereditary breast and ovarian cancer is associated with germ line mutations of *BRCA1* or *BRCA2* gene. Accordingly, MCT has been used successfully for uncovering otherwise hard or not to detect combined small deletion / duplication events (in the range of 3 to 17 kb) in *BRCA1* [44–46]. Also, *ѱ BRCA1* pseudogene and a before unknown 100-kb sequencing gap upstream of the *BRCA1* gene were identified by MCT. Even though more research studies with MCT concerning *BRCA1* gene were undertaken in between [47], a standard application in tumor genetic diagnostics was not established yet.

### MCT based studies of telomere length

Telomeres are specialized nucleoprotein structures at the ends of the linear chromosomes that function to protect the chromosome ends, thereby maintaining the stability of the genome. Telomeric DNA comprises repetitive sequences of the hexanucleotide TTAGGG_n_ repeat unit, bound in a sequence-specific manner to the protein complex shelterin, and assembled into macromolecular structures called telomere-loops (t-loops). In normal human somatic cells, telomeres range from 5–15 kb in length, and length variability was found for individual telomeres and different cell types. Inter-individual variability is also observed across the human population, superimposed to the well-established age-associated decline in telomere length [48]. Possibilities and advantages of MCT to check telomere length are summarized by Kahl et al. [49].

### MCT based diagnostics of viral integration

In terms of viral infection, the detection of foreign, viral DNA and its integration mode is intuitive and accurately possible by MCT. Especially, human papillomaviruses (HPVs) are frequently integrated in cancers. HPV genomes having a size of 7 to 8 kb, can be integrated as (type I) a single HPV genome, (type II) multiple, tandemly integrated HPV genomes, and (type III) multiple, tandemly integrated HPV genomes interspersed within host DNA [50]. Several MCT based studies for HPV integration [51–53] came to the conclusion that patients could benefit from this was of analyses, as subgroups based on viral integration sites could be established [53].

### MCT based studies of population specific polymorphisms

MCT can even be used to gain insights into population specific differences, yet suggested to be mainly polymorphic variations, which may in future be attributed to be associated also with susceptibilities to certain diseases. One study was on human blood neutrophil peptides (HNP1-3) and how copy number variants of alpha-defensins genes *DEFA1* and *DEFA3* vary and if they may be associated with infections and auto immune disorders [54]. In a second study analyzing CNVs of the human amylase gene clusters, MCT revealed unexpected genomic rearrangements leading finally to genomic instability, amplification and relocation of *AMY2A* and *AMY2B* genes. Here an association with obesity is suggested [55].

### MCT based research of DNA-replication

All afore mentioned applications are based on the GMC-type evaluation. Besides, MCT enables also combining GMC with a replication combing assay (RCA). Thus, DNA synthesis kinetics of a specific replicating sequence can be compared with the remainder replicating genome. Yet, there are many research studies in model systems like *Saccharomyces*, *Xenopus*, or human cancer cell lines published, accessing replication kinetics of mitochondrial DNA, fragile sites or telomeres (for review see [32]); however, no applications in clinical setting are available yet, and thus not topic of this review.

## Conclusions

MCT has great potential as an important cytogenomic tool in the field of chromosomic diagnostic and research [2]. Research applications of MCT mainly depend on research funds, which may be acquired more or less easily, if the underlying idea and project are of good quality. Introduction of MCT in diagnostics need to be at first approved by local authorities, like achieved for FSHD-diagnostics already; still the second big bottleneck is to find a way to get new methods into the national reimbursement catalogues. But, as MCT enable yet not, or by other means more complicated and more expensive cytogenomic approaches, there is to be expected a positive development.

## Data Availability

Data sharing is not applicable to this article as no datasets were generated or analyzed during the current study.

## Abbreviations

AFM: Atomic force microscopy
CE: Conformitè Européenne
cenM-FISH: Centromere-specific M-FISH
CO-FISH: Chromosome orientation-FISH
FAME: Familial adult myoclonic epilepsy
FISH: Fluorescence in situ hybridization
FM: Fluorescence microscopy
FSHD: Facioscapulohumeral muscular dystrophy
GMC: Genomic Morse code
HCM-FISH: Heterochromatin-oriented M-FISH
MCT: Molecular combing technology
MCB: Multicolor banding
M-FISH: Multicolor-FISH
pod-FISH: Parental origin determination FISH
Q-FISH: Quantitative FISH
RCA: Replication combing assay
SKY: Spectral karyotyping
